# State‐of‐the‐art microscopy to understand islets of Langerhans: what to expect next?

**DOI:** 10.1111/imcb.12450

**Published:** 2021-05-10

**Authors:** Pascal de Boer, Ben NG Giepmans

**Affiliations:** ^1^ Department of Biomedical Sciences of Cells and Systems University of Groningen University Medical Center Groningen Groningen The Netherlands

**Keywords:** biobank, data management and analysis, intravital microscopy, large‐scale electron microscopy, model systems, multimodal imaging

## Abstract

The discovery of Langerhans and microscopic description of islets in the pancreas were crucial steps in the discovery of insulin. Over the past 150 years, many discoveries in islet biology and type 1 diabetes have been made using powerful microscopic techniques. In the past decade, combination of new probes, animal and tissue models, application of new biosensors and automation of light and electron microscopic methods and other (sub)cellular imaging modalities have proven their potential in understanding the beta cell under (patho)physiological conditions. The imaging evolution, from fluorescent jellyfish to real‐time intravital functional imaging, the revolution in automation and data handling and the increased resolving power of analytical imaging techniques are now converging. Here, we review innovative approaches that address islet biology from new angles by studying cells and molecules at high spatiotemporal resolution and in live models. Broad implementation of these cellular imaging techniques will shed new light on cause/consequence of (mal)function in islets of Langerhans in the years to come.

## Graphical abstract

Innovative microscopic approaches that allow to address islet biology from new angles by studying cells and molecules at high spatiotemporal resolution and in live models are reviewed in this article. Broad implementation of these cellular imaging techniques will shed new light into the cause/consequence of (mal)function of islet of Langerhans in the years to come.

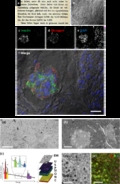

## INTRODUCTION

Islets of Langerhans have metabolic regulatory functions, most notably controlling blood glucose homeostasis by maintaining a balance between alpha cell‐secreted glucagon, which elevates glycemic levels, and beta cell‐secreted insulin, which decreases blood glucose. Type 1 diabetes (T1D) is caused by autoimmune destruction of beta cells, resulting in loss of functional secreted insulin levels that leads to constantly elevated blood glucose. Since Banting’s and colleagues first isolation of insulin and immediate successful clinical application 100 years ago, exogenous insulin administration remains the best therapy for T1D. Following Langerhans’ first description of islets, microscopy has been essential for understanding the structure, biology and microenvironment of beta cells and islets of Langerhans.

The history of discovery of the cellular composition of islets is well reviewed by Bonner‐Weir^1^ and by In’t Veld & Marichal.^2^ Here, we focus on the cutting‐edge microscopic techniques (being) developed over the past and future decades that may further help to understand islets of Langerhans and what may cause their dysfunction, especially that leading to T1D. The focus of this review is on techniques that greatly benefit from the evolved digital data infrastructure that can handle multimodal big data, which are used to identify molecules at (sub)cellular resolution. This includes identification of cells and molecules in fixed tissue, such as biobank material, high‐content multimodal electron microscopy (EM) and dynamic *in vivo* imaging of islets of Langerhans using state‐of‐the‐art probes and/or model systems. Several proof‐of‐concept studies showcasing the emerging techniques on islet imaging are discussed. Future broader implementation of these techniques holds great potential for better understanding islet biology in health.

## LIGHT MICROSCOPY: IDENTIFICATION OF CELLS AND MOLECULES IN FIXED TISSUE

For the discovery of islets and identification of molecules and cells, microscopic examination has been pivotal to unravel islet biology. Over the last decade, several of the routine techniques are being applied to biobanks and data are being shared. Moreover, analytical tools at the microscopic level help to identify players in islet (mal)function in high‐content and high‐throughput assays. In this section, we review the discovery of the first islet up to open‐access histochemical data sets of the pancreas.

### Discovery of islets of Langerhans

**Figure 1:**
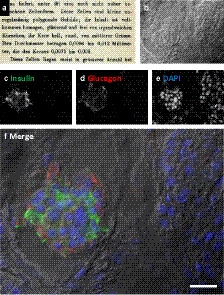
Identification of endocrine cells in human islets of Langerhans. **(a)** Script from Langerhans’ thesis (page 24^3^) describing the islets: “Diese Zellen liegen meist in grosserer Anzahl bei einander.” The description is continued on the next page of the thesis (see nanotomy.org for the full thesis) and based on light microscopic observations. After the development of **(b)** differential interference contrast and (immuno)fluorescence techniques as indicated here against **(c)** insulin (green) and **(d)** glucagon (red) with **(e)** a DNA counterstain (blue), which is merged in **(f)**,^4^ microscopic examination of islets has become straightforward. Scale bar: 0.02 mm. DAPI, 4′,6‐diamidino‐2‐phenylindole.

The microscopic discovery of cell clusters within the pancreas was by Paul Langerhans (Thesis, 1869): “Mostly, several cells are in close proximity, distributed peculiarly in the Parenchym of the gland (…) roundish clusters have a diameter of 0.1 to 0.24 mm” (Figure 1).^3^ Although he could not determine a specific function for these cell clusters, they were called islets of Langerhans by Edouard Laguesse in 1893 to acknowledge his discovery.^1^, ^2^ Moreover, Laguesse correctly described an endocrine function for the islets (Figure 1) of Langerhans for the first time. Five different endocrine cell types are present in the islets producing glucagon (alpha cells), insulin (beta cells), somatostatin (delta cells), pancreatic polypeptide (PP cells) and ghrelin (epsilon cells).^2^ The different endocrine cell types are not evenly distributed within islets of Langerhans and the cellular composition drastically differs among islets. Islets of Langerhans make up just a few percent of the complete pancreas.

### Immunohistochemistry and immunofluorescence

Microscopic inspection of human islets of Langerhans is performed on fixed tissue slices. Cell‐type discrimination requires immune labeling of the cell‐specific hormones (Figure 1).^4^ Spectral multiplexing determines the number of additional molecules of interest that can be costained, which is typically up to four or five different targets, including a nuclear stain with a DNA‐binding dye.^5^ Although immunolabeling is most routinely performed, some cell‐type‐specific histochemical stains can be applied.^2^ Fixed tissue slices are also the standard for (rodent) animal model pancreas tissue. In contrast to the human material, routine transgenic expression of genetically encoded tags in addition to immunolabeling is possible, which is, for example, useful for cell lineage tracing studies.^6^

### Biobanks of donors

The heterogeneity of islets within the pancreas, let alone between individuals, is enormous. Moreover, the pancreas is difficult to study in humans. Thanks to visionary initiatives, biobanks aiming to isolate pancreas from donors with diabetes, as well as controls, have been created. The Alan Foulis collection (or Exeter databank) focused on histopathological analysis of tissue sections,^7^ whereas the Network for Pancreatic Organ donors with Diabetes (nPOD) biobank^8^ and the Human Pancreatic Analysis Program (HPAP)^9^ optimally prepared donor tissue for a pleiotropy of different analysis methods, including analytical imaging, electron microscopic examination and even living slices as will be discussed later. New data‐sharing initiatives have accelerated data‐mining opportunities and thereby the usefulness of biobanks and their derived data.

### From biobanks to data repositories and data mining

A powerful addition of the biobanks is the metadata‐rich data repositories, such as digitalized pathology e‐slides from nPOD DataShare^10^ and HPAP,^9^the Pancreatlas with multimodal imaging data^11^ and large‐scale EM maps and analytical data via nanotomy.org by our group.^12^ These vast amounts of digitalized data are highly suitable for unbiased (automated) image analysis. Scripts to automatically identify immunolabeled islets of Langerhans are being created and shared.^13^, ^14^, ^15^ Moreover, image‐based machine learning algorithms to characterize the composition of altered pancreas in T1D are emerging.^16^ With the benefit of globally accessible universal and united data‐sharing capabilities, a generic gateway is being created to enable further data mining to help uncover potential clues that may help to find the trigger(s) leading to T1D in the future.

### Non‐optical analytical imaging approaches with (sub)cellular resolution

**Figure 2:**
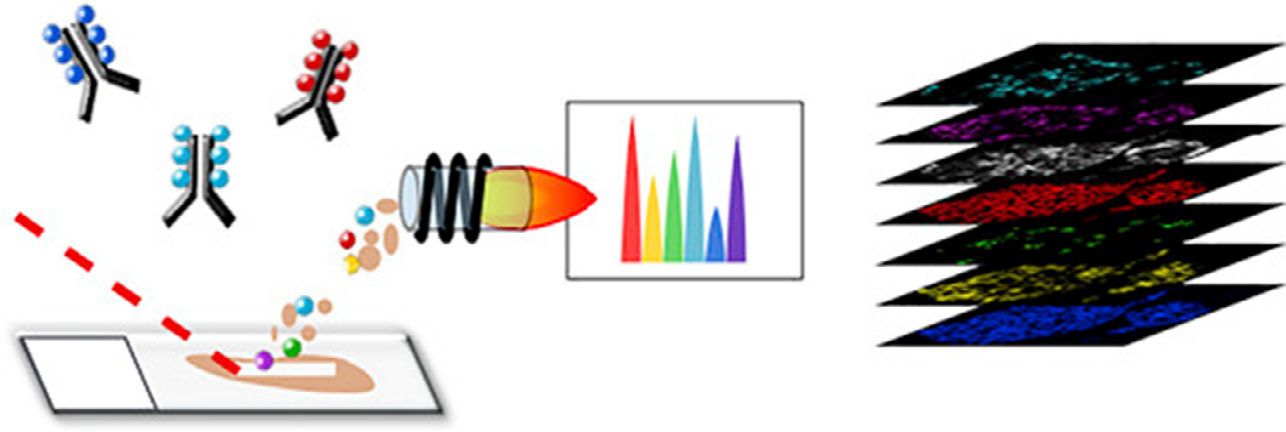
Imaging mass cytometry in islets of Langerhans. From left to right: tissue slices on object glasses are immunolabeled with primary antibodies conjugated with different metals. A laser ablates the tissue spot by spot, generating clouds of particles that are atomized and ionized before analysis by mass cytometry. Based on ion cloud composition, an image of the ablated area is created for each marker. Image and legend reprinted from Damond *et al*.^17^ with permission from Elsevier.

Immunofluorescent labeling approaches are very powerful to target and identify molecules with subcellular resolution. Identifying a multitude of different molecules simultaneously, however, is still limited because of spectral multiplexing. Although typically with lower resolution than immunofluorescence imaging, imaging mass cytometry (IMC) allows identification of tens of different targets simultaneously. IMC is based on labeling many protein targets with primary antibodies conjugated to unique metals on a microscopic section (Figure 2). Systemic laser ablation in a spot‐by‐spot fashion ionizes the sample and each metal is quantitatively measured. Following a complete run, each metal, and thus the corresponding antibody, and thus the target, for each spot is known. Typically, known targets to identify cells are used in combination with targets of yet‐unknown presence. Thus, each spot, with a lateral size much smaller than a single cell, contains information on abundance of tens of proteins. IMC has been elegantly applied to nPOD and HPAP biobanked material, allowing a pseudokinetics of islet destruction during T1D not only revealing the cells’ presence, but also their activity state based on expression of proteins^17^, ^18^ (Figure 2). IMC is sensitive, allows multiplexing and has subcellular resolution, but is focused on chosen proteins that are immunotargeted.

Mass spectroscopy imaging (MSI), which does not need the *a priori* choice of targets for immunodetection of, is an alternative approach to spatially define the presence of molecules. While MSI analysis is at lower resolution compared to IMC, the approach allows the discovery of molecules not specifically targeted and currently is maturing into a routine technique. Classical MS methods require tissue homogenization: spatial information on islets can be acquired by inititial selection and isolation of islets by, for example, laser capture microdissection.^19^ Spatial information is added via approaches that utilize MS as imaging modalities, such as matrix‐assisted laser desorption/ionization imaging mass spectrometry^20^ with a resolution in the order of tens of microns, or nanospray desorption electrospray ionization mass spectrometry^21^ with an approximate maximum resolution of 10 µm, thus at a single‐cell level. The molecular weight of detected fragments is analyzed per imaging spot, which may often be directly linked to molecular identity. Thus, the analysis goes beyond the proteome, which is a practical limit of most immuno‐based approaches described in the preceding sections, and includes metabolites and lipids.^20^ At present, proof‐of‐concept studies provide a data avalanche as many parameters per pixel are obtained. Non‐optical microscopy approaches have already shown their feasibility in identifying molecules in islet research and will continue to mature and help to analyze islet molecules in the years to come.

## ELECTRON MICROSCOPY: HIGHER RESOLUTION AND ULTRASTRUCTURE ANALYSIS

**Figure 3:**
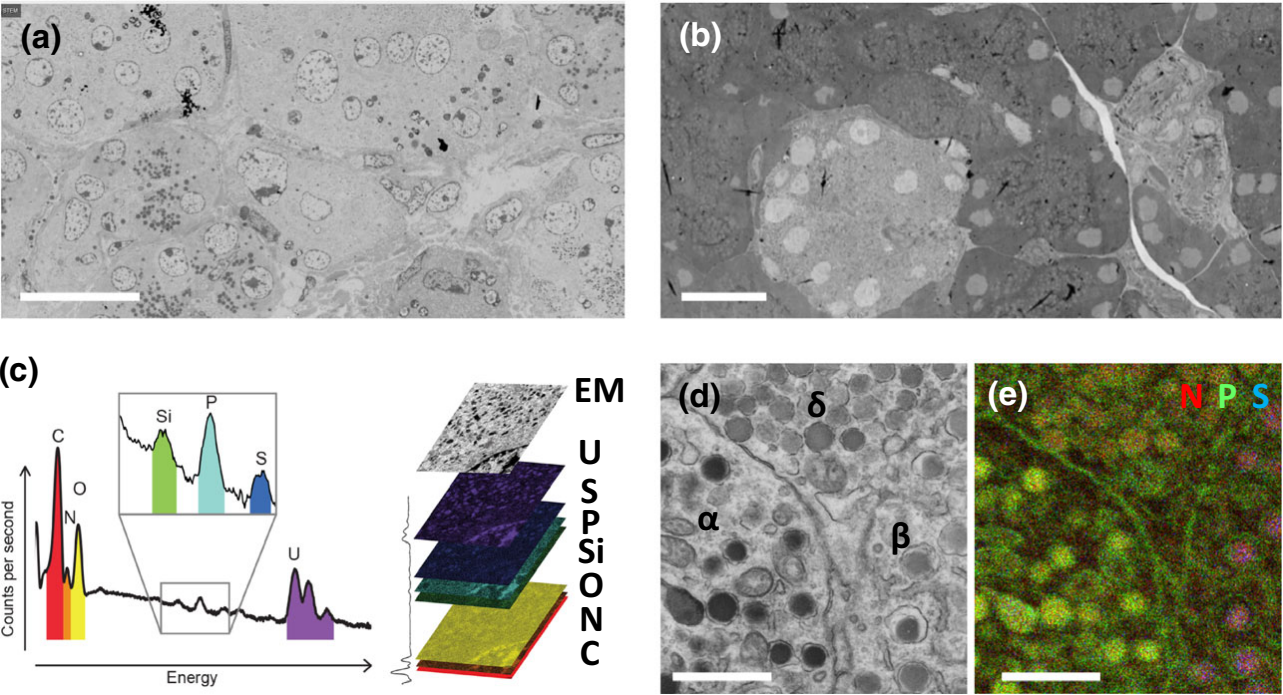
Large‐scale electron microscopy (EM) to analyze complete cross sections of islets at high resolution. **(a)** Routine “nanotomy” as performed in our imaging facility. A cross section of a human islet imaged at 2.5‐nm pixel size using a single‐beam microscope (total imaging time 18 h 40 min).^12^ **(b)** Multibeam nanotomy using a FAST‐EM prototype imaged at 4‐nm pixel size in 15 min. Images are produced by scanning all 64 beams simultaneously over a field of view, after which data are stitched together. Image courtesy Job Fermie, Delmic B.V., Delft, The Netherlands. **(c)** Adding an energy dispersive X‐rays (EDX) modality allows fingerprinting based on elements per pixel (left), which can then be combined with the EM image (right). **(d)** EM and **(e)** application of EDX in the analysis of islets of Langerhans. Nitrogen (N, red), phosphorus (P, green) and sulfur (S, blue) elemental maps are shown. The subcellular enrichment of certain elements aids in identification of distinct structures and organelles. Phospholipids in membranes contain high phosphorus, and therefore appear green. All vesicles with hormones are rich in nitrogen (amino acids), resulting in red somatostatin granules in delta cells (δ). The other endocrine cell types can be distinguished by enrichment of additional elements: insulin is rich in cysteines (sulfur), resulting in purple granules in beta cells (β). For yet unknown reasons, phosphorus is high in glucagon granules resulting in yellow granules in alpha cells (α).**(a**, **b)** Unpublished data, see for zoomable maps http://www.nanotomy.org; scale bars: 20 µm. **(c–e**) Reproduced from Pirozzi *et al*.^37^ **(c)** and Scotuzzi *et al*.^34^ **(d, e)**; scale bars: 1 µm.

Understanding T1D pathogenesis requires a complete insight into islets’ macromolecular composition and beta‐cell microenvironment. EM is used to study the cellular ultrastructure of fixed pancreas tissue with nanometer resolution. In EM, the different endocrine cell types of islets of Langerhans can be recognized based on secretory granule morphology. Whereas glucagon granules of alpha cells are dark, with a thin halo, insulin granules are lighter gray and mature granules have a crystalline core; somatostatin and PP secretory granules are homogenously light grey without any halo around the core^1^ (Figure 3).

### High‐content electron microscopy

Typical publication data of classical transmission EM are postage‐stamp‐sized images of high‐resolution ultrastructure with lateral fields of view of only a few micrometers. In the past decade, both 2D and 3D high‐content EM techniques expanded enormously to acquire nanometer‐resolution data of relatively large overviews and large volumes. Marsh and co‐workers expedited a flabbergasting tour‐de‐force to obtain 3D information on beta‐cell ultrastructure.^22^ Their efforts, only published 13 years ago, used an early digital camera, which was not routine in EM at that time. Focus was on imaging beta cells at high resolution in three dimensions using tomographic reconstruction (see Table 1 for an explanation of EM techniques). The enormous amount of data was probably the limiting step at that time, and therefore, only a few beta cells could be analyzed. Larger parts of islets could be examined by serial sectioning of consecutive thinner slices, although at lower resolution, to analyze more beta cells and has been instrumental to estimate beta‐cell life span based on lipofuscin body content.^23^ Higher voxel size resolution was achieved by serial block‐face EM,^24^ in which analysis of an enormous amount of insulin granules in different stages of maturation allowed reconstruction of the dynamics of granule maturation. At a slightly larger scale, this 3D technique, together with immunofluorescence identification of certain domain‐enriched protein, allowed identification of the interaction between the different hormone‐producing cells, as well as the vasculature, visualizing an outspoken beta‐cell polarity.^25^ Even higher axial resolution with focused ion beam milling serial scanning EM—now routinely with steps less than 20 nm and reaching macromolecular resolution—has been applied to study granule maturation.^26^

**Table 1:** Electron microscopy (EM) techniques in islet research

Method	Description	Reference
Tomography	Tilting of serial semithin 300‐nm sections. High resolution of low volume.	22
Serial sectioning	Serial ultrathin sections, with z‐resolution depending on section thickness. Large volumes.	23
3View	Block‐face imaging followed by automatic ultramicrotome‐assisted removal of imaged area. Volumes comparable to serial sectioning with improved z‐resolution.	24, 25
Focused ion beam milling serial scanning electron microscopy	Block‐face imaging followed by ion‐beam milling to remove imaged area. More precise, best z‐resolution, but limited lateral areas.	26
Nanotomy	Large‐scale 2D EM, higher throughput for individual samples, data sets are easily shared at nanotomy.org	12, 27 Figure 3a
Multi‐beam	In large‐scale microscopy, image acquisition has become a bottleneck. In scanning EM, the use of multiple beams speeds up acquisition by 1 or 2 orders of magnitude	30, 31 Figure 3b
ColorEM	Energy loss of the incident electrons and emission of X‐rays are used to analyze atomic composition	37

Acquisition time is being shortened by using sensitive cameras with lager pixel size or in scanning EM using multiple beams instead of a single beam.

### Large‐scale 2D cross‐sections: from biobanks to data repository

With the automation of electron microscopes and the capability of improved gigabyte data sets handling, “Google Earth”‐like large‐scale 2D imaging is feasible, and in our case it is routinely used to replace traditional single‐image EM^12^, ^27^ (Figure 3). The benefit of large‐scale EM includes the presentation of the overview of microscopic anatomy at low resolution, that can be easily compared with histology data, which can be zoomed onto nanometer‐scale resolution; therefore, we introduced the term nano‐anatomy or nanotomy^28^ (Figure 3a, follow the link in the legend to navigate through the data at high resolution; Table 1). Recently, a constantly expanding open‐access repository of nPOD samples was created. Ultrastructural analysis of donor material is now just a mouse click away.^12^ The information‐dense character of EM data makes it valuable for reuse by others^29^ and thereby such databases prevent the need for extensive and time‐consuming EM sample preparation and data‐acquisition procedures. While large‐scale EM is not yet used routinely by many others, because the acquisition rate is typically long (overnight), further automation and faster microscopes such as the recently developed and commercially available multibeam EMs  will take nanotomy to the next level (Figure 3b; Table 1).^30^, ^31^

### Analytical electron microscopy: “ColorEM”

EM reveals cellular ultrastructure, and analysis of data is typically by morphological characteristics of cells, organelles and macromolecules, including in the nanotomy data described above. Immunolabeling to identify proteins has been highly instrumental in identifying cells and hormones in islets of Langerhans. While for light microscopy immunolabeling is very straightforward, immunolabeling on EM samples is very limited because of conflicting sample preparation requirements. However, protocol optimization has enabled immuno‐EM labeling of insulin,^32^, ^33^ glucagon^34^ and somatostatin^35^ for correlative light and EM, which allows fluorescence guidance of gray‐scaled EM samples. More recently, elemental analysis is becoming a routine application in the life sciences, helping identify structures in EM micrographs. In addition to the analysis of electrons, now the loss of electron energy^36^ or energy of emitted X‐rays from the sample is also determined (Figure 3c–e).^34^ Thus, adding multimodality helps to identify molecules or structures in EM maps, that is, the differentiation between granules or cell types^12^ is guided by adding information from the additional detector typically in false color, and therefore popularly called “ColorEM” (Figure 3c–e; Table 1).^37^ ColorEM data mining of the nPOD nanotomy repository for zoomable EM maps^12^ revealed an increased cellular colocalization of both exocrine and endocrine secretory granules in autoantibody‐positive and T1D donor samples accompanied by aberrant ultrastructural morphology, which might indicate an exocrine pancreatic involvement in T1D etiopathology.

### Data analysis

High‐content EM techniques result in an avalanche of gray‐scaled data which are hard, if not impossible, to completely analyze manually. Combination with multimodal imaging is currently the best solution to identify molecules and structures of interest. Although both correlative light and EM and colorEM are very powerful and protocols are improving for broader implementation, targeting and sensitivity issues remain. Therefore, as with plugins for analysis of scanned histochemistry slides discussed above, automated EM analysis would be the next goal. Although important efforts with respect to insulin granule recognition have been made,^38^ automated recognition of very heterogeneous EM data is far from straightforward. Important steps are currently being made to develop machine learning‐based image analysis.^39^ These workflows are mostly pioneered on brain tissue; therefore, the long‐term goal would be to broadly implement deep learning in EM islet research.^40^

## INTRAVITAL MICROSCOPY

The imaging methods discussed thus far, immunohistochemistry and immunofluorescence of pancreatic tissue slices and EM, are performed on fixed tissue and therefore they are all endpoint measurements. In the next section, tools, systems and models to image dynamical processes in islets and beta cells are discussed, which could provide much needed insights into the cause–consequence questions in the context of T1D pathology. The development and implementation of genetically encoded fluorescent proteins, such as the green fluorescent protein (GFP), have revolutionized biology: the study of molecules in living systems. Fluorescent techniques, probes and model systems currently converge to allow researchers to address functional processes and role of molecules in living organisms.

### Probes and sensors to study the islet of Langerhans

Externally introduced probes enable labeling and (fluorescently) visualization of molecules of interest and many different kinds of probes exist from immune‐targeting to genetically encoded tags.^41^ In addition to static immunolabeling, probes that allow spatiotemporal imaging of beta‐cell function and presence have been developed which include fluorescent proteins and small molecules.

**Figure 4:**
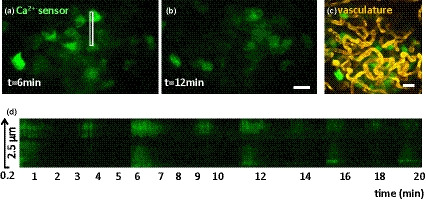
Longitudinal intravital analysis of islets of Langerhans. A cutting‐edge experiment by combination of several techniques highlighted in this review. Using a glass window in mice, well illustrated elsewhere,^52^ and a genetically encoded sensor that changes fluorescence upon binding of calcium ions introduced by injection of viral particles for transduction for selective expression in beta cells since the control is by the insulin promotor which is imaged using two‐photon excitation.**(a, b)** Two still images of the time lapse show the spatiotemporal fluctuation of the calcium sensor signal in the islet beta cells. **(c)** An intravenously injected fluorescence molecule highlights the vasculature of the islet. **(d)** Kymograph of every 6 s per frame of the boxed area in **a**. Note the different oscillation frequency of the top and bottom cell. Scale bars: 20 µm. Bottom: x/y (2.5 µm × 0.2 µm). Click for the movie link. Reproduced with permission from Reissaus *et al*.^52^

Probing molecules via fluorescent proteins have numerous benefits: plasmids can be designed and created that carry specific targets under the control of cell‐specific promotors, for example, insulin promoter ensuring expression in beta cells only of c‐peptide fused to GFP.^42^ In addition, several means of controlled delivery to living systems are available, such as using viral transduction of *ex vivo* (human) islets or other genetic tools to create transgenic animal models.^42^, ^43^, ^44^, ^45^ During the past 25 years, starting with GFP, fluorescent protein technology flourished, encompassing hundreds of fluorescent proteins with different properties. Based on these fluorescent proteins as well as on small molecules, many sensors for bioprocesses have been developed and are easily shared within the scientific community, for example, via www.addgene.org. These functional sensors change their fluorescent protein properties in different biological settings (e.g. pH, oxidative stress, chelation) or when other neighboring fluorescent proteins influence the fluorescent properties.^41^ Dozens of these probes have been applied to study islet biology and are presented in a recent comprehensive review.^46^ For instance, fluorescently labeled ligands specific for beta cells (e.g. exendin‐4 conjugates) can target glucagon‐like peptide receptor 1 (GLP1R) to monitor beta‐cell mass.^47^ More recently, a 30‐amino‐acid‐derived peptide of exendin, which does not automatically activate the receptor, was used in combination with small fluorescent dyes well suited for either intravital microscopy or super‐resolution microscopy. These small “LUXendin” dyes target the cell surface and can therefore be simply applied or injected.^48^ Another target is the zinc ion: zinc is present in certain secretory granules and is co‐released during exocytosis which can be measured with small‐molecule sensors that become highly fluorescent upon zinc binding to study the secretory mechanism in living systems. Recently, a functional sensor for secretory granule zinc content allowed both analysis of trafficking of secretory granules and discrimination of different endocrine cell types.^49^ Contrary to applying exogenous probes, employing the autofluorescence properties of NAD(P)H via fluorescence lifetime imaging has been established as a method to directly image islet metabolism.^50^, ^51^ An example of application of functional dyes to monitor reactive oxygen levels (stress), as well as calcium fluxes for beta‐cell function, is presented by Linnemann and co‐workers, using genetically encoded sensors transferred by viral transduction. Transplanted islet physiology and *in vivo* islet physiology can be studied in mice having a transplanted imaging window, as will be detailed later^52^ (Figure 4). Fluorescent sensors are typically used in parallel with spectral different fluorophores to obtain additional information; for example, injection of fluorescent dextran to visualize the vasculature, or highlighting specific immune cells through immunolabeling of certain CD cell‐surface molecules. The tremendous advancements in probe and sensor toolbox is very timely and matches the technical progress in live‐cell and intravital microscopy and model systems to study islet biology.

### Techniques: from standard confocal, to two‐photon, toward light sheet microscopy

What a great time to be a microscopist! The revolution in computer sciences and engineering in optical systems, including cameras and multimodal microscopy, has accelerated the development of microscopes for cellular imaging—and beyond—in the past decade. In fixed specimens, analytical imaging techniques allow multilabeling of dozens of protein targets or the transcriptome, or chemical analysis of the sample with high spatiotemporal precision, down to the macromolecular level using colorEM. Although data mining will be a challenge using these techniques, data‐sharing repositories are popping up to allow reuse of data.

Most studies on fluorescent imaging of islets to identify presence of molecules, islet mass and islet function use a form of 3D imaging. 3D reconstruction of islet architecture in relation to vascularization and innervation using fluorescent probes can be studied as exemplified by several informative 3D reconstructions,^53^ with results emphasizing the heterogeneity of islets.^54^ Confocal laser scanning microscopy and spinning disk laser scanning microscopy allow optional sectioning by specifically gathering emitted light of fluorophores from the focal plane. A disadvantage of live‐cell imaging is the high‐intensity laser beam, and the exclusion of photons that are not in the focal plane. These photons outside the focal plane still may damage the fluorophore and/or sample. In multiphoton microscopy, the excitation volume determines where fluorophores are being imaged. The emitted light will only be collected from the small excited volume, so it is collected more efficiently. Using an infrared pulsed laser, deeper penetration and nonlinear phenomena, such as second harmonic generation, are used by many for intravital imaging. A main ongoing development for much faster imaging is light‐sheet microscopy: specimens are illuminated from the side and all data are collected from the emitted photons in that single light sheet; especially for sub‐millimeter specimens, including isolated islets and zebrafish larvae in combination with the fluorescent tools, light sheet microscopy will be the revolutionary live‐cell imaging technique widely applied in the next decade. The imaging speed makes light sheet a promising method to include in high‐throughput microscopy and screening. Given the heterogeneity of islets of Langerhans within a single pancreas, as well as between individuals and between species,^54^ these imaging techniques that allow building of databases will better enable understanding of normal physiology and the multiple causes of islet destruction.

### Live model systems

Crucial for primary validation of probes and sensors and new techniques are cell lines and isolated islets. However, in this review, we focus on *in vivo* imaging of complete islets of Langerhans in tissue. This varies from human pancreatic slices and human transplanted islets to intravital microscopy in mice and zebrafish larvae.

#### Anterior chamber of the eye

The anterior chamber of the eye (ACE; explained and illustrated in detail by Nilsson *et al*.^55^) has been pioneered by the Berggren group as a transplantation site for islets that subsequently can be followed over time in mice. ACE was introduced by Speier *et al*.,^56^ where transplantation of hundreds of islets led to reversal of hypoglycemia in streptozotocin‐treated recipient mice. The transplanted islets could well be monitored using two‐photon microscopy for up to 4 months using GFP expression in the transplanted islets, in addition to the visualization of vasculature through injected Texas‐Red‐labeled dextran.^56^ Furthermore, label‐free optical coherence microscopy allowed visualization of microvascularization and dynamic blood flow of ACE‐transplanted islets.^57^ The model is not only valuable to study the interaction of transplanted islets with the vasculature, but also the immune response can be analyzed over time, making use of fluorescent immunolabeling of specific immune cells. Moreover, ACE islet transplantation is suitable for real‐time imaging of beta‐cell dynamics.^58^ While probes may be introduced *ex vivo* in the transplanted islets, or prelabeled in, for instance, immune cells, even endogenous functional characteristics can be monitored, such as NADPH autofluorescence to monitor glucose metabolism or application of third harmonics generation to analyze collagen in living mice.^59^

#### Slices

Isolated islets are being widely used to better understand islet biology. Modification, for instance, by viral transfer of genetically encoded reporters, is very feasible, as in the aforementioned example.^52^ However, the isolation procedure itself will induce cellular stress, and islets are no longer in contact with their surrounding exocrine cells. To study islets within the context of exocrine pancreas, dedicated mouse models to create pancreatic slices have been developed.^60^, ^61^ Recently, human material derived from the nPOD infrastructure has allowed 10‐day‐long studies on beta‐cell fate in human pancreas slices.^62^

#### Mouse: Externalized pancreas

The ability to image islets in the pancreas in living animals would circumvent any isolation‐ or *in vitro*‐induced alterations. *In vivo* imaging of mouse pancreas has been performed by abdominal surgery to expose the pancreas to the coverslip.^63^, ^64^ This model can, for example, be used to study glucose‐regulated blood flow in mouse islets via imaging of fluorescent dextran and transgenic fluorescently labeled endocrine cells,^64^ and *in vivo* immune cell–islet interaction with cellular resolution using multiphoton excitation.^63^, ^65^ Likewise, using a faster recording with a spinning disc confocal microscope, fast single granule exocytosis events can be monitored in intact pancreas using GFP‐based fluorescent reporters. The imaging setup allows monitoring of flashes of exocytosis caused by a pH‐dependent increase in probe fluorescence upon granule fusion, where the probe concentration—and hence the localized signal—rapidly drops when secreted.^42^ As opposed to the terminal studies discussed here, mice with an implanted window allow longitudinal imaging of islets over several weeks.

#### Mouse window: Longitudinal

Transplanted islets can be followed using the ACE model described above, but a more realistic transplantation site being explored for therapy is under the kidney capsule. However, functional recovery by islet transplantation in patients is only transient. Basic questions such as how the functionality and acceptance of islets, including vascularization, need to be understood in detail to improve islet transplantation endeavors. An imaging window surgically introduced in the skin of mice allows tracking of transplanted human islets for up to 2 weeks, again using two‐photon microscopy and properly introduced fluorescent probes.^66^ A similar window was used in a 31‐day study focusing on the pancreas at the other side of the window to visualize *in vivo* viral transduction of beta cells with fluorescent biosensors for calcium dynamics (Figure 4) and reactive oxygen species.^52^

The next step for *in vivo* imaging may be to modulate cell physiology using optogenetics, as has been demonstrated by the activation of a genetically engineered channel rhodopsin that, when stimulated with light, causes a rise in cytoplasmic calcium, which is a preceding step in exocytosis. Expression of such a sensor in beta cells, and precise spatiotemporal light activation, allows modulation of beta‐cell physiology *in vivo*, enabling light‐controlled insulin release.^67^

#### Zebrafish

The zebrafish model system has been widely used to study vertebrate development. Already at 48 h after fertilization, the first islet is formed with endocrine cells as identified by immunostaining.^68^ In these early studies two decades ago, the authors rightfully mentioned that genetic studies would be very valuable to understanding pancreas development. Zebrafish larvae are optically transparent; therefore, zebrafish models allow study of islet biology intravitally and noninvasively with both the vasculature and innervation present without any surgical procedure. A subset of these studies in zebrafish, which are now booming in *in vivo* islet research, includes the application of fluorescent proteins to identify cells including lineage tracing to determine cell origin,^69^ autoimmune attack^70^ and cell‐specific chemical‐ or optogenetic‐mediated ablations.^71^, ^72^, ^73^ Genetic engineering of zebrafish is straightforward, thus enabling the application of the aforementioned probes to monitor calcium fluxes^74^, ^75^, ^76^ and reactive oxygen species‐induced injury.^77^ Of course, the appropriate (custom) probes or biosensors and microscopic screening devices need to be properly devised.

While not yet widely used, our expectations of using zebrafish models to study islet stress or immunological responses upon many different triggers are sky high: The larvae can be easily produced in high numbers, and therefore are very suitable for high‐throughput screens, such as small‐molecule library screening for compounds that have an impact on endocrine cell (trans)differentiation or cell function,^78^, ^79^ or increase beta cell mass.^80^ Moreover, intact living zebrafish are very suitable for the emerging fast light‐sheet imaging method discussed above.

## Future outlook

The imaging evolution, from fluorescent jelly fish to intravital functional imaging, and the revolution in automation and (biobank) data handling and microscopic techniques as well as increased resolving power of analytical imaging techniques are now converging. This is well illustrated in two recent examples: intravital imaging of different dynamic parameters in beta cells described by Reissaus *et al*.^52^ (Figure 4) and intravital imaging combined with advanced EM methods for novel insights into paracrine delta cell functions.^81^ Broad implementation of these cellular imaging techniques will shed new light into the cause–consequence relations of islet of Langerhans (mal)function in the context of T1D pathology in the years to come.

## CONFLICT OF INTEREST

The authors declare that no relevant conflicts of interest exist.

## Author Contribution

**Pascal de Boer:** Conceptualization; Data curation; Investigation; Methodology; Writing‐original draft; Writing‐review & editing. **Ben NG Giepmans:** Conceptualization; Funding acquisition; Methodology; Supervision; Visualization; Writing‐original draft; Writing‐review & editing.

## Data Availability

The data that support the findings of this study are openly available in nanotomy at www.nanotomy.org ^3^ ; Zoomable maps of data presented in Figure 3 are accessible online.
